# Editorial Notes

**Published:** 1911-06

**Authors:** 


### Editorial Notes.

**Sickness and Invalidity Bill, 1911.**

No medical journal can be silent on this question; the outcry on all hands is acute. It is said that it is too soon to cry out, but probably it will be useless to do so later: it must be now or never. The doctors are said to be dissatisfied with the present proposals, and no wonder. It would be intolerable to perpetuate the abuses of the present club system in a greatly extended Government scheme. Medical men naturally will object to their work being almost entirely through the Friendly Societies; they object to a capitation contract per annum; they claim that the public shall have a free choice of doctor, and that the payment should be by visit. They claim also that a uniform rate of payment is not reasonable, that many extras should be possible, and that a special advisory committee will be necessary to adjudicate on these, and that, further, a special Arbitration Board for the settlement of disputes will be essential.

We have to thank Dr. Harry Gray for the following criticism on the present aspects of things, but these vary from week to week, and will be much discussed before the Government scheme becomes more concrete.

" The Government Sickness and Invalidity Bill introduced in the House of Commons on May 4th deserves the close attention of all medical men. A close scrutiny of its provisions for medical attendance might lead one to imagine that the Chancellor of the Exchequer had relied on the altruism of the medical profession to over-rule consideration of its own interests. The average general practitioner, at any rate, is so cognisant of the blessings which sick and invalidity insurance will put within reach of all classes of the community that it requires some fortitude to raise a dissentient voice amid the general chorus of praise and congratulation.

" The effect of the Bill on medical practice may be considered in two directions. First, financially, will the profession benefit ? In so far as some millions of persons who have hitherto gone without medical attendance, or obtained it without paying for it, will now become contributors to the medical income, the profession stands to gain both work and pay. Some financial gain should also result from the doctor's being relieved of the cost of providing drugs. The Chancellor will no doubt consider this a makeweight for the small premium which he proposes should be raised from 4s. (which he takes as the usual) to 5s. The prospective gain here, however, is chimerical. In the first place, the average 4s. capitation grant is the nominal and not the real rate. In most Friendly Societies there is generally a minority of members who pay for the club doctor, but employ private practitioners, so that if the nominal rate is taken as 4s. the real rate will vary from 4s. 6d. to 7s. 6d. Free choice of doctor, which the profession is inclined to insist on, makes the nominal rate the real one. Further, instead of gain, there is likely to be loss in the conversion of the club member into a State protégé, inasmuch as the medical services required by the State will probably be greater than those generally given under the contract system. This is the weak point also in the British Medical Association's Provident Service, which takes no account of this lowering of remuneration and increase of service entailed by admitting the principle of free choice of doctor. Since on the credit side we must place the conversion of some non-payers into payers, we must place on the debit side the fact that several millions (the entire population if it so wills, including the Chancellor himself) who have hitherto paid the doctor ordinary or even good fees will become club patients at the contract rate. To those practitioners whose practice consists entirely, or almost entirely, of contract work, and whose position is rather that of whole-time salaried officers, the State Bill may make practically no difference. The gain of 1s. per head and relief from the cost of drugs will be discounted by the extra attendance required, and it is likely that their actual income and work will remain the same, owing to the defections brought about by freedom of choice of doctor.

"In the case of other practitioners, the financial gain or loss will depend chiefly on the proportion of their club to private work. The larger the number of their paying private patients converted into State beneficiaries at a capitation rate, the greater will be their loss ; whereas in a practice consisting almost entirely of clubs and poor patients, with a large proportion of bad debts, some relief may accrue from the operation of the National Insurance Scheme.

" More important, however, than the question of financial gain or loss is that of the effect likely to be produced on the conditions of practice, and the outlook here is still more unpromising. There has been a noticeable tendency in recent years to minimise the inherent defects of the capitation system ; the ' battle of the clubs ' has become chronic, and its interest wanes ; the increasing stress of competition, making any kind of ' appointment ' appear desirable, has placed the question of remuneration in such a prominent position as to make one forget that there were, and always must be, other disabilities of practice under the capitation system. The essence of the club contract is insurance. With everyone obtaining medical service on the club system the old relation between doctor and patient, of helper and helped, is modified by the new bond of insurer and insured ; for it is not the State which will insure the beneficiary against the cost of medical services, but the doctor who becomes an underwriter or one-man insurance company. It is inevitable that the relation of insurer and insured should tinge the dealings of doctor and patient : a scrutiny on the one hand as to the services demanded in return for the premium offered, and on the other as to the service received for the premium paid. This has in time past been the bane of club practice, and it is difficult to see how it can be got rid of in the new regime.

" It may be some little advantage to the profession in the way of preventing overstocking, that with universal club service the possible income of the medical practitioner will be very accurately defined. The available patients in the country divided among the present number of doctors gives to each a clientèle of 1,400 ; 1,400 patients at 5s. per head means a possible income of £350 a year gross. In rural districts, with a motor-car or even a modest horse turn-out, this leaves but a beggarly income for a professional man, and it is likely that when the return for capital and brains is so accurately defined, there will be a still further falling-off in the entries at the medical schools. This, indeed, is the only hope for the profession of medicine—that economic laws will bring just retribution upon the attempt to sweat a learned profession. But even if ground may eventually be regained in this direction, the blow to the old position of the doctor as a family friend will never be recovered from. A new *régime* opens before us. What standing we shall have under it twenty years hence it is impossible to forecast."

**Laryngological Section of the Royal Society of Medicine.**

A LARGE and distinguished gathering of specialists in this branch of medicine journeyed to Bristol on Friday, June 2nd, from London and the provinces at the invitation of the President (Dr. Watson Williams), to attend a meeting of the section held by the kindness of the Council of the University and Professor Francis, in the new Chemical Wing. Much interest was shown by the visitors in the size and general excellence of arrangement of our new buildings.

An extensive and interesting programme was arranged, embracing demonstrations of methods of diagnosis and treatment, exhibition of cases and specimens, and a discussion on a subject of common interest to the rhinologist and ophthalmologist. Those who saw the demonstration of bronchoscopy by Dr. Watson Williams for the first time must have been surprised to see with what ease and certainty the direct examination of the lower air passages can be carried out by the experienced.

Although direct examination of the œsophagus has now been in use for a considerable period, it is only recently that a satisfactory method of gastroscopy has been perfected, so that a demonstration by Drs. Hill and Herschell on the living subject of the method which they have devised caused a great deal of interest. By means of an instrument on the plan of a cystoscope passed through a modified Killian's tube, they are able to inspect the whole extent of the gastric mucosa, and those of us who had the opportunity of seeing this demonstration will agree that the view obtained leaves little to desire. In skilled hands this method should prove a real advance in the diagnosis of gastric conditions.

Dr. Finzi also demonstrated on a patient the method of applying radium to a carcinomatous gullet, and the results hitherto published seem to show that radium so applied can produce alleviation of symptoms, and sometimes perhaps even cure.

Dr. Finzi and Mr. C. F. Walters also showed a case of great interest, in which an inoperable tumour of the cheek (probably endothelioma) had disappeared under the application of radium. Among other cases shown was a series showing the result of the President's osteoplastic frontal sinus operation, on the results of which he was heartily congratulated. During the meeting which followed the exhibition of cases and demonstrations, the President handed to Sir Felix Semon a volume commemorating the formation of a Semon Lectureship in Laryngology at London University, founded with a sum of money collected from colleagues and friends of that distinguished laryngologist on his retirement from active practice.

The meeting ended with a discussion on "The relationship of diseases of the nose and accessory sinuses to affections of the eye and orbit."

The subject was introduced by Dr. St. Clair Thomson with a general outline, illustrated by lantern slides, of the anatomical relations involved and of patients showing some of the conditions mentioned.

Mr. Richardson Cross followed, presenting the subject from the ophthalmologist's point of view. The discussion then became general, and though somewhat curtailed owing to the lateness of the hour, the general consensus of opinion seemed to be that the nasal cavities have not till now received their due attention as a cause of ocular and orbital disease.

The social side of the meeting was as successful as the scientific. The President and Mrs. Watson Williams entertained the Council of the Section and other friends at dinner, and afterwards received the members of the Section and a large number of guests at the Spa.

On Saturday a visit was made to Bath, a luncheon being given to the visitors by Mr. and Miss Pagan Lowe at the Guildhall, followed by an inspection of the Baths. The visit ended with a reception at the Roman Baths by the Mayor and Mayoress of Bath.

